# Caveolin-1 Impacts on TGF-β Regulation of Metabolic Gene Signatures in Hepatocytes

**DOI:** 10.3389/fphys.2019.01606

**Published:** 2020-01-31

**Authors:** Mei Han, Zeribe Chike Nwosu, Weronika Piorońska, Matthias Philip Ebert, Steven Dooley, Christoph Meyer

**Affiliations:** ^1^Section Molecular Hepatology, Department of Medicine II, Medical Faculty Mannheim, Heidelberg University, Mannheim, Germany; ^2^Department of Internal Medicine, The Second Hospital of Dalian Medical University, Dalian, China

**Keywords:** caveolin-1, transforming growth factor beta, metabolism, liver diseases, microarray

## Abstract

Caveolin-1 (CAV1) is a membrane protein associated with metabolism in various cell types. The transforming growth factor beta (TGF-β) is a pro-fibrogenic cytokine in the liver, but its metabolic gene signatures remain unclear to date. We have previously shown that CAV1 alters TGF-β signaling and blocks its pro-apoptotic function. Here, we defined TGF-β-induced metabolic gene signatures in hepatocytes and assessed whether CAV1 abundance affects TGF-β control of those metabolic genes. Microarray analyses of primary hepatocytes after TGF-β stimulation (48 h) showed differential expression of 4224 genes, of which 721 are metabolic genes (adjusted *p* < 0.001). Functional annotation analysis revealed that TGF-β mainly suppresses metabolic gene network, including genes involved in glutathione, cholesterol, fatty acid, and amino acid metabolism. TGF-β also upregulated several genes related to glycan metabolism and ion transport. In contrast to TGF-β effects, CAV1 knockdown triggered the upregulation of metabolic genes. Immortalized mouse hepatocytes (AML12 cells) were used to validate the gene changes induced by TGF-β stimulation and CAV1 knockdown. Noteworthy, of the TGF-β metabolic target genes, CAV1 modulated the expression of 228 (27%). In conclusion, we present several novel metabolic gene signatures of TGF-β in hepatocytes and show that CAV1 abundance alters almost a third of these genes. These findings could enable a better understanding of TGF-β function in normal and diseased liver especially where differential CAV1 level is implicated.

## Introduction

Transforming growth factor beta is the best-studied member of the TGF-β superfamily, which also includes activins, inhibins, and BMPs (Weiss and Attisano, 2013). TGF-β triggers signaling by binding to its cell surface serine/threonine-specific protein kinase receptors (TGFBR I–III). The activated receptor complexes mediate the phosphorylation of R-Smads (i.e., Smad2 and Smad3) that subsequently mediate downstream activities by shuttling into the nucleus to regulate gene transcription. Besides this canonical pathway, TGF-β/receptor complexes can transduce signaling via non-canonical pathways, e.g., ERK, PI3K/AKT, p38, and JNK signaling pathways (Attisano and Wrana, 2002; Weiss and Attisano, 2013).

Transforming growth factor beta is known to play various roles in different cellular contexts, including regulation of growth, apoptosis, differentiation, inflammation, and epithelial–mesenchymal transition (Massague, 2012; Fabregat et al., 2016), as well as fibrosis in diverse diseases. Several reports underline the importance of hepatocyte-specific TGF-β signaling. For example, TGF-β triggers apoptosis of hepatocytes by interacting and cooperating with factors like FasL, TNF-α, NFκB, and inducting reactive oxygen species (Meyer et al., 2013c). In the study from Seki et al., the authors illustrated that TGF-β signaling in hepatocytes drives fibrogenesis, inflammation, and subsequently HCC development in mice with hepatocyte-specific Tak1 deficiency. This was mainly due to increased hepatocyte apoptosis, which could be rescued by Tak1/TGF-β receptor II hepatocyte-specific double KO (Yang et al., 2013). It has previously been observed that TGF-β participates in all phases of liver disease development, including early stages like inflammation, steatosis, and fibrosis to advanced stages, such as cirrhosis and HCC (Dooley and Ten Dijke, 2012). In addition, TGF-β has been already associated with metabolic deregulation. TGF-β increases glucose transporter 1 expression in breast cancer cells and hexokinase 2 in fibroblasts (Li et al., 2010; Hu et al., 2014), therewith modulating glycolysis. Yang et al. (2014) described that TGF-β signaling induces lipogenesis-associated genes and suppresses β-oxidation-associated factors to promote lipid accumulation in hepatocytes upon palmitate supplementation *in vitro*. *In vivo*, Smad3-deficient mice fed a HFD were resistant to obesity and hepatic steatosis, which illustrated that the TGF-β/Smad3 signaling pathway plays a crucial role in regulating energy homeostasis (Yadav et al., 2011). However, the metabolic gene signatures of TGF-β in liver parenchymal cells have not been clearly defined yet.

Caveolin-1 is a membrane protein forming omega-shaped invaginations termed caveolae. Previous studies have shown that CAV1 is a regulator of diverse metabolic processes. CAV1 deletion downregulated lipid metabolism in mouse embryonic fibroblast primary cultures, livers, and adipose tissues from CAV1 global KO mice as demonstrated by genomic profiling (Fernandez-Rojo et al., 2013). In addition, CAV1 KO mice were shown to have massively increased levels of free fatty acids and triglycerides, and were resistant to HFD-induced obesity (Razani et al., 2002).

Besides these functions, CAV1 is a potent regulator of various signaling pathways and affects TGF-β signaling via diverse mechanisms (Meyer et al., 2013b). For example, CAV1 expression suppresses the TGF-β signaling cascade by interacting with the TGF-β receptor I or via regulating TGF-β type II receptor gene expression in NIH3T3 fibroblast cells (Razani et al., 2001; Lee et al., 2007). *In vivo*, CAV1 scaffolding domain peptides were proven to dampen liver fibrosis by inhibiting TGF-β/Smad signaling (Lu et al., 2018). Also, lower CAV1 expression in hepatocytes increased TGF-β-mediated apoptosis (Meyer et al., 2013c). In this study, we explored the notion that CAV1 level affects TGF-β control of metabolism and assessed metabolic gene signatures of TGF-β signaling activation in hepatic cells.

## Materials and Methods

### Mice

Mice were purchased from Janvier labs (C57BL/6). Animals were fed standard chow and water *ad libitum*. All animal procedures were performed according to national and international guidelines. Prior to experimentation, ethics approval was obtained from the local ethics committee of Baden-Württemberg.

### Primary Hepatocyte Isolation and Cultivation

Primary hepatocytes were prepared from 12-week-old C57BL/6 mice via the two-step collagenase (C2-22, Merck Biochrom) perfusion method as previously described (Godoy et al., 2009). Then, isolated hepatocytes were plated on 6-well plates and cultured in Williams E medium (F1225, Merck Biochrom) with 1% L-glutamine, 1% P/S, 0.1% dexamethasone, and 10% FBS. After hepatocyte attachment (∼4 h), culture medium was changed to starvation medium (no dexamethasone and FBS).

### AML12 Cell Culture

AML12 murine hepatocyte cells were cultured in DMEM/F-12 (21331020, Thermo Fisher Scientific) with 10% FBS, 1% glutamine, 1% P/S, 0.5% insulin–transferrin–selenium (ITS, Gibco), and 0.1% dexamethasone (D1159-100MG, Sigma-Aldrich) in a humidified atmosphere containing 5% CO_2_ at 37°C. Hanks’ balanced salt solution (HBSS, Sigma-Aldrich, H6648) was used for cell washing steps.

### siRNA Transfection

After attachment, primary hepatocytes were cultured overnight with 10 nM siRNA targeting CAV1 (siCAV1, Dharmacon, Thermo Fisher Scientific, United States) and negative control siRNA (siCon, SI03650318, Qiagen) using RNAiMAX transfection reagent (Invitrogen, Darmstadt, Germany) according to the manufacturer’s instructions. Next day, the hepatocytes were treated with 5 ng/ml recombinant TGF-β1 (Peprotech, Hamburg, Germany) and cultured additional 48 h in starvation medium. For AML12 cells, 10 nM siCon and siCAV1 was transfected to the cells with Lipofectamine RNAiMAX reagent after attachment, and incubated for 24 h. Afterward, cells were cultured in starvation medium for 12 h, and subsequently treated with 5 ng/ml recombinant TGF-β1 for 24 h.

### RNA Isolation, cDNA Synthesis, and qPCR

Total RNA was isolated using InviTrap Spin Universal RNA Mini Kit from all sample groups according to the manufacturer’s instruction (Stratec Biomedical AG, Germany). One microgram of RNA was converted to cDNA using RevertAid H Minus Reverse Transcriptase (EP0451, Thermo Scientific, Germany). Gene transcript levels were quantitatively determined by EvaGreen qPCR Mix Plus (Solis BioDyne, Tartu, Estonia) on the AB StepOnePlus. Quantitative polymerase chain reaction (qPCR) analysis was performed in a 10-μl total reaction volume with a reaction mix as 2 μl of EvaGreen, 12.5 ng of cDNA, and 10 nM of gene primers. Transcript levels of each gene were normalized to the level of PPIA (*Ppia*), and experiments were performed in triplicate (or as indicated). Primers used in this experiment were from Eurofins (Germany). The gene primer sequences used were as follows: *Cav1*: forward: GAA GGG ACA CAC AGT TTC GAC, reverse: GGA TGC CGA AGA TCG TAG ACA. *Ppia*: forward: GAG CTG TTT GCA GAC AAA GTT, reverse: CCC TGG CAC ATG AAT CCT GG. Other primer sequences used in this study are listed in Table 1.

**TABLE 1:** Primer sequences.

Mouse primers	Forward (5′–3′)	Reverse (5′–3′)
*Aldh1a1*	CAAGGCCAATGTTGTGTCGC	GCTCGCTCAACACTCCTTTTC
*Ampd3*	AAACAACTGACCTGTCCTCCC	TCAGCCCGTTAGGAGAATGG
*Aqp8*	CTGGGGAGCAGACACCAATG	CAGGAGCCCAGTATTCGGAC
*Asns*	GACTCTAAGGTGGGAAGCGG	CACTCAGACACTGCACGGAA
*Chst8*	TCAGAGCAGTGGTGTCTACTC	ACCCCATACAGCCTTCACAGA
*Cyp4b1*	TATCCTATGCACCAGCAGCG	TGGGGTACAGGTGGGTAGAG
*Dhdh*	AGCAGAAGTTCGGGAGATGG	GGCTCCAAATGGCCTCCATA
*Gpx3*	GGGCTTCCCTTCCAACCAAT	ATTAGGCACAAAGCCCCCAC
*Gsta4*	ACTTTAATGGCAGGGGACGG	TACTTGGCCGAAAAGCAGGT
*Gsto1*	AGACCTCGTGCTTCCAGAGT	GATTCTCCCGACATCGCTGA
*Hsd11b2*	TCTGGAAATCACCAAGGCCC	AGTTCCACGTCAGCCACTAC
*Psat1*	GCTGTCGCCTTAGCACCA	ATGCCGAGTCCTCTGTAGTC
*Ptgs2*	TGCTGGTGGAAAAACCTCGT	GGTGCTCGGCTTCCAGTATT
*Slc25a37*	ACGCCATGTATTTTGCCTGC	ACTCCCAGCTACCCCATTAG
*Slco3a1*	GCACACCAGACACATAGGACA	TTCAGCCCTCCTGTAGTCAC
*Smad7*	GGCCGGATCTCAGGCATTC	TTGGGTATCTGGAGTAAGGAGG
*Tap1*	GCGGCAACCTTGTCTCATTC	CATGTTTGAGGGTGCCAACG

### Western Blot

Cell lysates were extracted by RIPA buffer and centrifuged at 2000 × *g* for 10 min. Protein concentration was assessed with the Bio-Rad protein assay kit according to the manufacturer’s instructions and quantified via absorbance measurement at 690 nm. Equal amounts of protein (30 μg) were loaded to 12% sodium dodecyl sulfate polyacrylamide electrophoresis (SDS-PAGE) gels and blotted onto 0.2 μm Nitrocellulose membranes (Carl Roth, Germany). Membranes were blocked with 5% BSA in TBST at room temperature for 1 h, and incubated overnight at 4°C with primary antibodies. Primary antibodies against CAV1 (T3267, Cell Signaling), pSmad3 (ab52903, abcam), and β-actin (sc47778, Santa Cruz) were used. HRP-linked anti-mouse (sc-2005, Santa Cruz) and anti-rabbit antibodies (sc-2357, Santa Cruz) were applied as secondary antibodies. Signals were visualized by incubating the blots in Supersignal Ultra solution (Pierce, Hamburg, Germany) and subsequent imaging with the Fusion SL4 device.

### Microarray Analysis

Total RNA was isolated from hepatocytes that were transfected with siCon or siCAV1 overnight and subsequently with or without TGF-β treatment for 48 h. Acceptable RNA quality was confirmed by capillary electrophoresis on an Agilent 2100 bioanalyzer (Agilent). Gene expression profiling was performed using arrays of the mouse MoGene 2.0 type (Affymetrix). Biotinylated antisense cRNA was then prepared according to the Affymetrix standard labeling protocol with the GeneChip WT Plus Reagent Kit and the GeneChip Hybridization, Wash and Stain Kit (both from Affymetrix, Santa Clara, United States). Afterward, the hybridization on the chip was performed on a GeneChip Hybridization oven 640. Dying took place in the GeneChip Fluidics Station 450, and thereafter, chips were scanned with a GeneChip Scanner 3000. The entire equipment used was from the Affymetrix company (High Wycombe, United Kingdom). Microarray data were deposited in GEO^1^ database (GSE137339).

### Pathway and Other Data Analyses

The microarray dataset GSE35431, comparing livers from global CAV1 KO mice and wild-type littermates, was downloaded from the GEO website. In this dataset, genes with a *p-*value < 0.05 were selected as dysregulated genes, and ranked by logFC (fold change). Subsequently, they were used for functional annotation and gene ontology analyses using DAVID Bioinformatics Resources 6.8^2^. Overlapping genes were visualized by Venny tools^3^.

### Statistics

Analyses were performed using GraphPad Prism version 6.0 Software. Two-way ANOVA was applied for qPCR data and Tukey’s *post hoc* test was followed. Statistical significance was indicated as follows: ^∗^*p* < 0.05; ^∗∗^*p* < 0.01; ^∗∗∗^*p* < 0.001, and ^*⁣*⁣**^p < 0.0001.

## Results

### CAV1 Knockdown Upregulates Metabolic Genes in Hepatocytes

**FIGURE 1:**
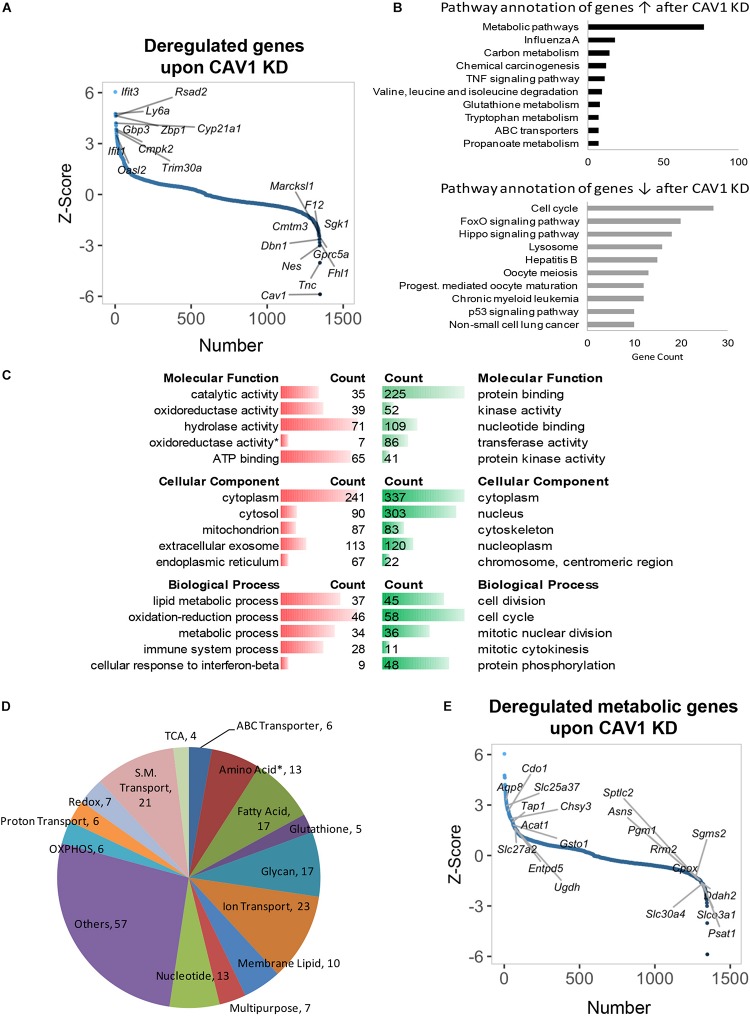
Gene expression profiling and deregulated metabolic genes upon CAV1 knockdown in primary hepatocytes after 48 h. **(A)** Distribution of altered genes by *Z*-score (*N* = 1348, highlighted top 10 upregulated and top 10 downregulated genes). **(B)** KEGG pathway annotation of total deregulated genes indicated that CAV1 knockdown caused upregulation of metabolic processes. **(C)** Molecular functions, biological processes, and cellular component analyses of total deregulated genes. **(D)** Classification of 229 deregulated metabolic genes in biochemical metabolic processes. **(E)** Distribution of deregulated genes by *Z*-score (*N* = 1348, highlighted metabolic top 10 upregulated and top 10 downregulated genes). Data from each analyzed group was from a mixture of three mice. ↑, upregulated; ↓, downregulated; red, upregulated; green, downregulated. ^∗^, all amino acids besides glutamine and serine.

Caveolin-1 has been associated with lipid metabolism, altered mitochondrial function, and energy balance in mouse hepatocyte (Asterholm et al., 2012; Fernandez-Rojo et al., 2012, 2013; Fernandez-Rojo and Ramm, 2016; Nwosu et al., 2016; Kuo et al., 2018). However, the overall role of CAV1 in cell metabolic fate is still poorly understood. Thus, we knocked down CAV1 in freshly isolated mouse hepatocytes and performed microarray analysis of gene changes. We identified 1348 genes that were differentially expressed after 48 h CAV1 knockdown. Topmost among these were *Tnc*, *Nes*, *Fhl1*, *Sgk1*, and *Cav1* (all downregulated), and *Ifit1/3*, *Ly6a*, *Zbp1*, *Cyp21a1*, and *Trim30a* (all upregulated) (Figure 1A and Supplementary Table S1). Pathway analysis of the differentially expressed genes showed that CAV1 knockdown caused overall upregulation of metabolic processes and the downregulation of cancer-related pathways (Figure 1B). Among the involved metabolic processes were glutathione (e.g., *Mgst3*, *Gss*, *Gclc*) and carbon metabolism (e.g., *Acadm*, *Pgd*, *Sdsl*; Supplementary Table S2) as well as valine, leucine, and isoleucine degradation (e.g., *Hsd17b10*, *Acadm*, *Mcee*; Supplementary Table S2). Further, many metabolic alterations related to oxido-reductase activity, mitochondria, and lipid metabolic processes emerged in gene ontology analysis of upregulated genes. In contrast, the downregulated genes were mostly associated with protein binding, nuclear activities, and cell cycle functions (Figure 1C). Assessment of an independent dataset from *Cav1-/-* mouse livers (Asterholm et al., 2012) confirmed that the majority of metabolic processes were induced *in vivo* (Supplementary Figures S1a–d).

Of the 1348 deregulated targets upon CAV1 knockdown in our dataset, we identified 229 metabolic genes (Supplementary Table S3). These included several targets in amino acid, fatty acid, glycan, and nucleotide metabolism; OXPHOS; ion/small molecule transporters; and other multifunctional metabolic genes (Figure 1D). Specifically, upregulated metabolic genes belonged mainly to fatty acid metabolism (e.g., *Acat1/2*, *Mttp*, and *Acss2*), OXPHOS (e.g., *Cox15/18*, *Uqcrc1*, and *Ndufb8*), and small-molecule transporters (e.g., *Aqp8*, *Slc25a37*, and *Scl27a2*, Figure 1E and Supplementary Table S4). In contrast, we observed a clear pattern of downregulated genes in proton transporters (e.g., *Atp6v1h*, *Atp6v1c1*, and *Atp6v0b*), glycan metabolism genes (e.g., *Idua*, *Fuca2*, and *Gusb*), as well as a mixed expression pattern for amino acid metabolic genes (including upregulated genes, e.g., *Cdo1*, *Sephs2*, and *Kmo*, and downregulated genes, e.g., *Psat1*, *Asns*, and *Sephs1*, Figure 1E and Supplementary Table S4). Of note, there was little overlap between the *in vitro* expression data and *in vivo* CAV1 KO mouse livers previously published by Asterholm et al. (2012). Only 18 metabolic genes emerged as having consistent expression patterns (i.e., up- or downregulated in both datasets, *p* < 0.05), and those included *Cps1* in urea cycle, Oat (which utilizes urea cycle intermediates in glutamate synthesis), *Pcyt2*, *Pla2g12b*, *Srxn1*, *Nudt9*, *Mlycd*, *Decr2*, *Pigp*, *Stard10*, and *Cyb5r3* (upregulated), and *Dsel*, *Kcnk5*, *Gba*, *Impad1*, *Inpp5a*, *Ndst1*, and *Slc46a1* (downregulated, Supplementary Figure S1e). Overall, these data reveal that CAV1 impacts on a plethora of yet unappreciated metabolic processes in hepatocytes.

### TGF-β Stimulation Suppresses Metabolic Genes and Reveals Core Signatures in Hepatocytes

**FIGURE 2:**
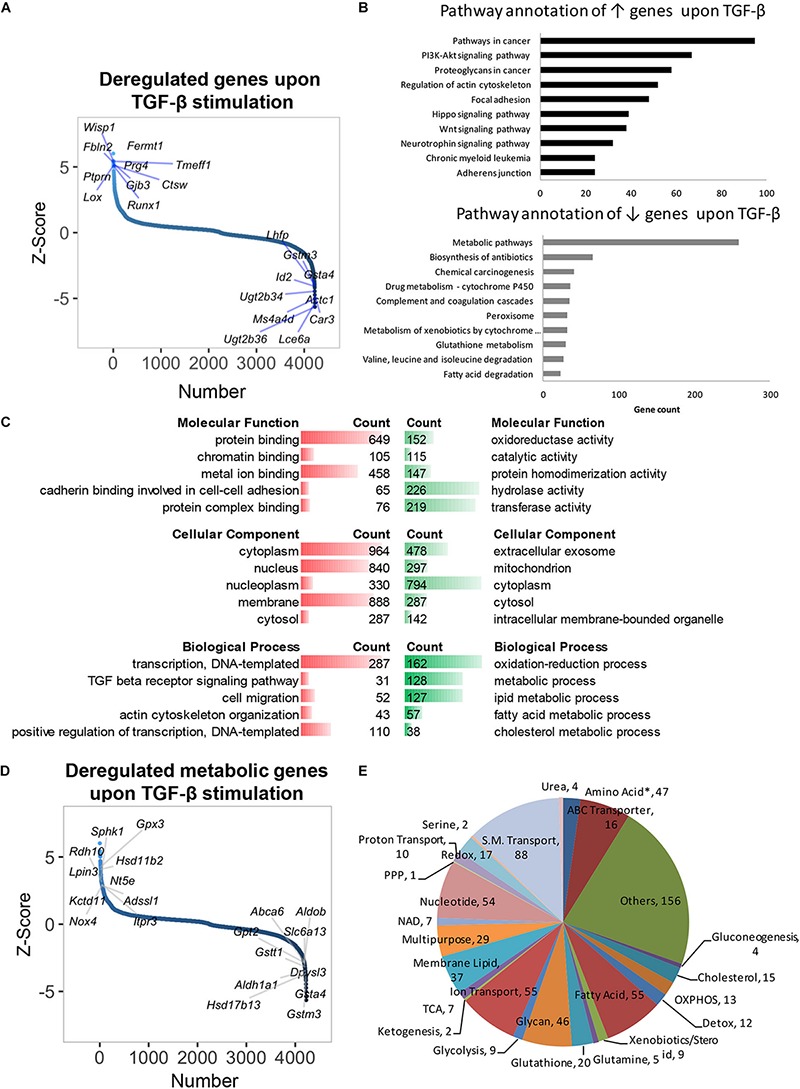
Gene expression profile and deregulated metabolic genes of primary hepatocytes treated with TGF-β for 48 h. **(A)** Distribution of altered genes by *Z*-score (*N* = 4224, highlighted top 10 up- and top 10 downregulated genes). **(B)** KEGG pathway annotation of total upregulated or downregulated genes indicated that TGF-β stimulation suppressed metabolic genes. **(C)** Molecular functions, biological processes, and cellular component analyses of total deregulated genes. **(D)** Distribution of deregulated genes by *Z*-score (*N* = 4224, highlighted metabolic top 10 upregulated and top 10 downregulated genes). **(E)** Classification of 721 deregulated metabolic genes in biochemical metabolic processes. ↑, upregulated; ↓, downregulated; red, upregulated; green, downregulated. ^∗^, all amino acids besides glutamine and serine.

As earlier mentioned, TGF-β is known to mediate various activities in hepatocytes (e.g., pro-inflammatory effects, epithelial–mesenchymal transition, senescence, apoptosis), but its control of metabolic processes beyond lipid metabolism is largely unexplored to date. TGF-β stimulation altered 4224 genes after 48 h (*p* < 0.05, Figure 2A and Supplementary Table S5). Contrary to the effect of CAV1 knockdown, pathway enrichment analysis showed that genes perturbed by TGF-β were involved in oncogenic processes, notably in PI3K-Akt (e.g., *Hsp90ab1*, *Pdgfb*, and *Pdgfa*), Hippo (e.g., *Mob1a*, *Bmpr2*, and *Itgb2*), and Wnt signaling (e.g., *Camk2g*, *Tcf7l2*, and *Tcf7l1*, Figure 2B and Supplementary Table S6). As expected, we also observed the induction of known TGF-β signaling-regulated genes, e.g., *Tgif1*, *Tgfb1*, *Tgfbr1*, *Tgfbr2*, *Bmpr1a*, *Bmpr2*, *Bambi*, *Acvr1*, *Cdkn2b*, *Smad4*, *5*, *6*, and *7* (Supplementary Figure S2a). Besides these, TGF-β prominently induced, e.g., *Fermt1*, *Wisp1*, and *Prg4*, and suppressed genes such as *Ugt2b36*, *Lce6a*, and *Ms4a4d* (Figure 2A), thus revealing yet unknown signatures of TGF-β.

Gene ontology and pathway annotation revealed significant suppression of metabolism (Figure 2C). Specifically, TGF-β profoundly suppressed genes in glutathione metabolism (e.g., *Gclc*, *Srm*, and *Anpep*), fatty acid degradation (e.g., *Cpt1c*, *Acaa2*, and *Cpt1b*), and xenobiotics/drug metabolism (e.g., *Cyp2f2*, *Ugt2b1*, and *Gstm6*, Figure 2B and Supplementary Table S6). A total of 721 metabolic genes were altered by TGF-β in normal hepatocytes (*p* < 0.05), with 237 being upregulated (e.g., *Gpx3*, *Hsd11b2*, *Sphk1*, *Rdh10*, *Lpin3*) and 484 being downregulated (e.g., *Gsta4*, *Gstm3*, *Hsd17b13*, *Aldh1a1*, and *Gstt1*, Figure 2D and Supplementary Table S7). The differential genes were mainly involved in fatty acid, amino acid, glycan, and nucleotide metabolism, as well as ion/small molecule transporters (Figure 2E and Supplementary Table S8). TGF-β has been associated with lipid metabolism (Yang et al., 2014), and indeed we found a notable regulation of lipid metabolic genes in our pathway annotation and gene ontology analysis (Supplementary Table S9). Further, we overlapped our dataset with findings from a prior study where TGF-β signatures in mouse primary hepatocytes were assessed at early (0.5–2 h) and late time points [4–24 h (Coulouarn et al., 2008)]. Intriguingly, of the 34 overlapping genes from both datasets, 33 were from late time points, while only 1 gene (*Aldh3a2*) was suppressed at an early time point (Supplementary Figure S2b). When comparing overlapping upregulated genes, only five metabolic genes (i.e., *Rdh10*, *Gsto1*, *Slc6a8*, *Sult1e1*, and *Lpgat1*) from late time points and seven metabolic genes (i.e., *Slc25a37*, *Slc23a2*, *Clic4*, *Slc20a1*, *Qcnt2*, *Slc29a1*, and *Coq10b*, Supplementary Figure S2c) from early time points were found to be similarly regulated. Taken together, our findings indicate a profound suppression of metabolic genes by TGF-β in hepatocytes at later time points, which, to date, including physiologic consequences, has not been sufficiently appreciated.

### CAV1 Influences TGF-β Metabolic Gene Signatures

**FIGURE 3:**
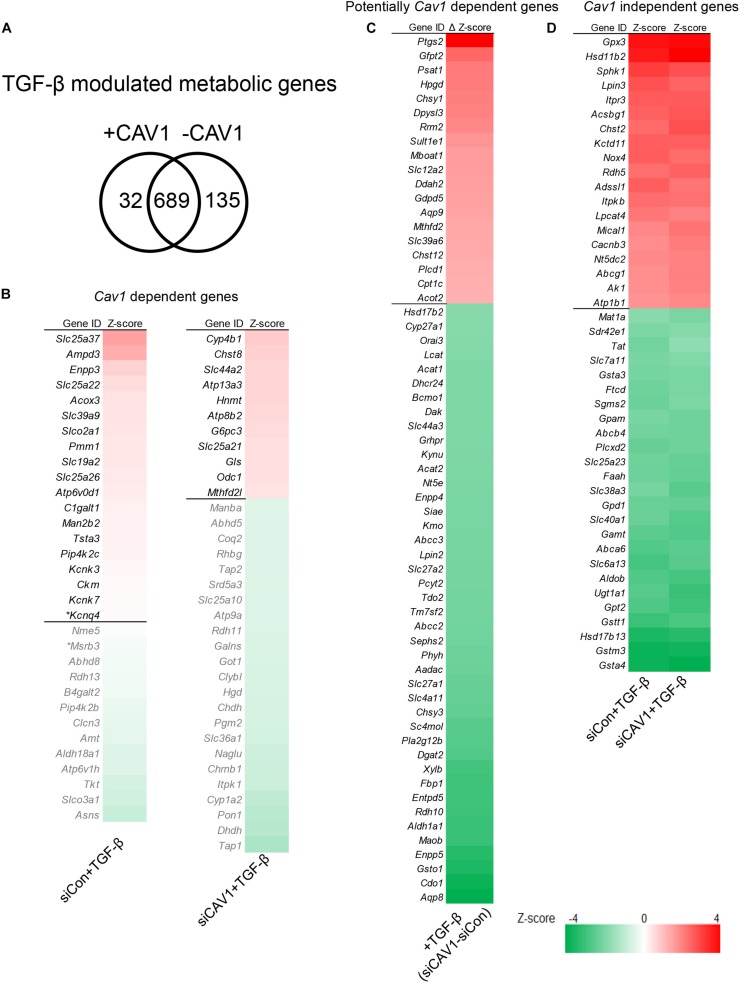
TGF-β control of metabolic gene signatures was influenced by CAV1. **(A)** Venn diagram of TGF-β modulated metabolic genes in normal CAV1 expression and after CAV1 knockdown. **(B)** CAV1-dependent TGF-β regulated genes: Left – 32 genes altered in normal CAV1 expression dataset, *p < 0.05, others *p* < 0.01. Right – genes altered in CAV1 knockdown dataset (34 genes with a threshold of *Z*-score > ±0.5). **(C)** Potentially CAV1-dependent TGF-β regulated genes: 19 upregulated genes with a threshold of Δ*Z*-score > 0.5 between siCon and siCAV1 datasets; 42 downregulated genes with a threshold of Δ*Z*-score < –0.5 between siCon and siCAV1 datasets. **(D)** CAV1-independent TGF-β regulated genes: 19 upregulated genes regulated by TGF-β in both normal CAV1 expression and knockdown datasets with a threshold of average *Z*-score > 2.0; 25 downregulated genes in both normal CAV1 expression and knockdown datasets, with a threshold of average *Z*-score < –2.0. siCon, control siRNA; siCAV1, siRNA targeting CAV1.

A CAV1–TGF-β crosstalk has not yet been particularly explored in the context of metabolic gene signatures. We compared the genomic profile of TGF-β stimulation in control hepatocytes and after knocking down CAV1 (Figure 3A). Across both conditions, TGF-β stimulation altered a total of 856 metabolic genes (*p* < 0.05, Supplementary Table S7). Of those, 32 were altered in the presence of CAV1, whereas 135 (19.4%) were altered only in the CAV1 knockdown hepatocytes (Figure 3B and Supplementary Table S7), implying these CAV1-dependent TGF-β regulated metabolic genes. Further, 689 genes were differentially expressed both in the normal and CAV1 knockdown hepatocytes (Supplementary Table S7). Interestingly, the pattern of TGF-β-induced gene expression, i.e., either up- or downregulated, was identical for 99% of the perturbed metabolic genes in the CAV1 knockdown and control hepatocytes (except for *Ace2*, *Scn3a*, *S1c39a6*, *Trpm1*, *Aqp5*, and *D2hgdh*). With the 689 overlapping genes, we further explored the possibility that CAV1 abundance affects the degree of TGF-β impact on the metabolic genes. To approach this question, we calculated differences in expression (*Z*-score) between the TGF-β target genes from CAV1-treated knockdown and wild-type hepatocytes. Using a *Z*-score of ±0.5 at which most of the genes had a *p* < 0.01, we observed that upon CAV1 knockdown, 19 genes were significantly more upregulated by TGF-β (e.g., *Ptgs2*, *Gfpt2*, and *Psat1*), whereas 42 genes were more downregulated by TGF-β (e.g., *Aqp8*, *Cdo1*, and *Gsto1*, Figure 3C and Supplementary Table S10). We designated these genes (in total 61) as potentially CAV1-dependent genes and classified the remaining 628 genes as CAV1-independent genes. Besides these, we identified >40 other strongly changed genes (*Z*-score ± 2, Figure 3D and Supplementary Table S10).

### Validation of TGF-β Target Genes in AML12

**FIGURE 4:**
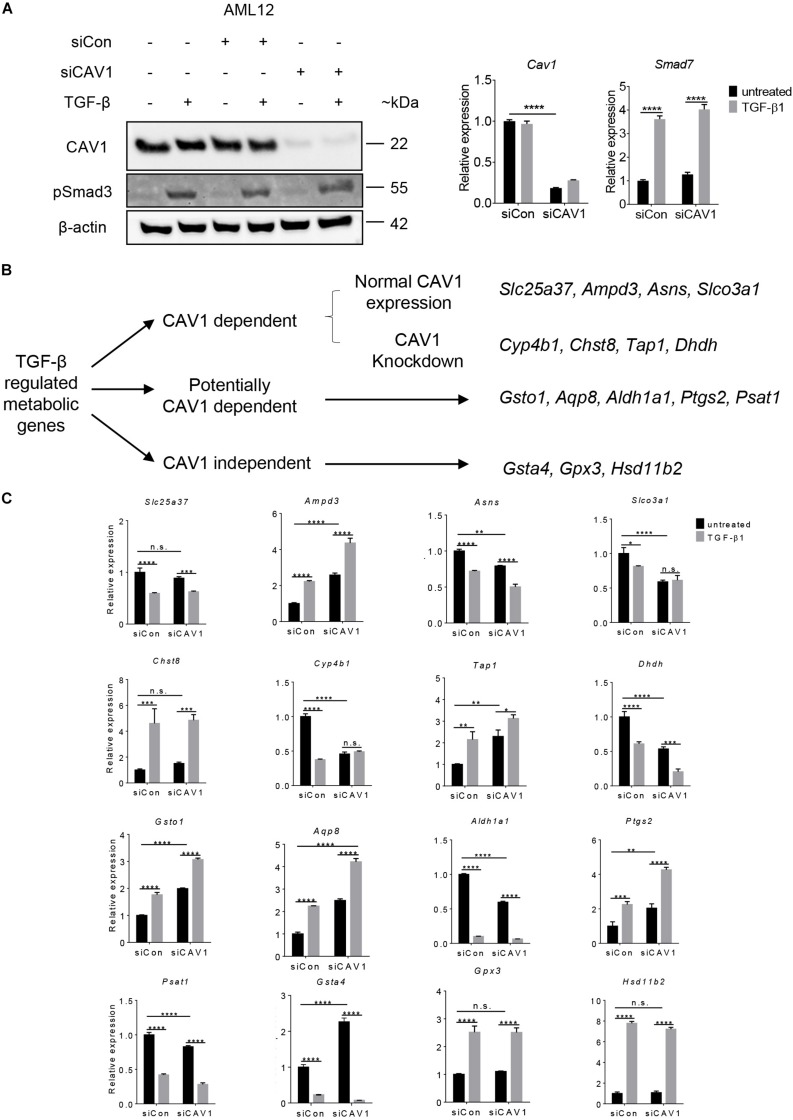
Validation of TGF-β regulated genes in AML12 cells. **(A)** Western blot (CAV1 and pSmad3 expression) and mRNA expression analyses for *Cav1* (for validating knockdown) and TGF-β target gene *Smad7* (for verifying TGF-β signaling activity), respectively. **(B)** Clustering scheme of the analyzed 16 metabolic candidate genes altered by TGF-β stimulation in the context of CAV1 abundance (*N* = 3). **(C)** mRNA expression of the 16 metabolic genes in AML12 upon CAV1 knockdown and TGF-β stimulation for 24 h. Statistical analysis was done by two-way ANOVA followed by Tukey’s *post hoc* test. siCon, control siRNA; siCAV1, siRNA targeting CAV1. *p < 0.05; **p < 0.01; ***p < 0.001; ****p < 0.0001.

To validate the TGF-β effect on metabolic gene regulation and considering the impact of CAV1, we used an immortalized mouse hepatocyte cell line, AML12 cells. CAV1 knockdown and TGF-β signaling activation were confirmed at the protein and mRNA level, by measuring CAV1 and the TGF-β components pSMAD3 and *Smad7*, respectively (Figure 4A). We next compiled a list of 16 metabolic genes altered by TGF-β in hepatocytes. This list consisted of the two topmost upregulated and downregulated metabolic genes in CAV1-expressing (WT) hepatocytes (i.e., *Slc25a37*, *Ampd3*, *Asns*, and *Slco3a1*), the four most differentially regulated genes (two upregulated, two downregulated) in CAV1 knockdown hepatocytes (i.e., *Cyp4b1*, *Chst8*, *Tap1*, and *Dhdh*), as well as the strongest regulated genes in both datasets (i.e., up: *Gpx3*, *Hsd11b2*; down: *Gsta4*). Also included were five candidates (e.g., *Gsto1*, *Aqp8*, *Ptgs2*, *Psat1*, and *Aldh1a1*) regulated by TGF-β in both datasets, but also impacted by CAV1 expression levels (Figure 4B). mRNA expression analyses showed that all 16 genes were significantly regulated by TGF-β in AML12 cells (5 ng/ml for 24 h, Figure 4C). Among them, 12 genes were regulated as expected from hepatocyte microarray data, including *Ampd3*, *Asns*, *Slco3a1*, *Chst8*, *Dhdh*, *Gsta4*, *Aldh1a1*, *Gpx3*, *Hsd11b2*, *Gsto1*, *Ptgs2*, and *Psat1*, whereas four genes were regulated in the opposite direction (i.e., *Slc25a37*, *Cyp4b1*, *Tap1*, and *Aqp8*). Glutathione peroxidase 3 (*Gpx3*) and *Hsd11b2* showed CAV1-independent regulation, as predicted. Similarly, *Ampd3*, *Slco3a1*, *Asns*, *Cyp4b1*, and *Dhdh*, which were predicted as CAV1 dependent, also showed a consistent expression pattern as predicted from microarrays. Importantly, we confirmed that the genes predicted to be potentially CAV1 dependent, e.g., *Gsto1*, *Aqp8*, *Ptgs2*, *Psat1*, and *Aldh1a1*, were indeed CAV1-dependent. To summarize, of the 16 selected targets, 12 genes were regulated by TGF-β the same way as observed in hepatocytes, and among those 12 genes in AML12, 10 genes (*Gpx3*, *Hsd11b2*, *Gsto1*, *Psat1*, *Ptgs2*, *Aldh1a1 Ampd3*, *Slco3a1*, *Asns*, and *Dhdh*) showed CAV1 dependency as in primary hepatocytes, therewith supporting the validity of primary cell data. Interestingly, all five genes that were beforehand defined as potentially CAV1 dependent (from the primary hepatocytes dataset) proved to be CAV1-dependent genes in the AML12 cell line as well (Figure 4C). The 61 potentially CAV1 dependent genes were therefore defined as CAV1 dependent, and in summary, 228 genes (∼27%) of TGF-β regulated metabolic genes were CAV1 dependent. We therefore conclude that CAV1 exerts a crucial and previously unappreciated impact on TGF-β-mediated control of metabolic processes in hepatocytes.

## Discussion

Caveolin-1 has been reported to be an important regulator of lipid accumulation, glucose metabolism, and mitochondrial integrity (Fernandez-Rojo and Ramm, 2016), suggesting that CAV1 has a potent influence on diverse metabolic processes. Furthermore, CAV1 is capable of triggering autophagy in normal and cancer cells. However, some observations regarding CAV1 function were inconsistent across independent studies, indicating a context-dependent role in distinct cell types (Nwosu et al., 2016). For example, in colorectal cancer, increased CAV1 expression can promote glucose uptake via stimulating glucose transporter 3 transcription (Ha et al., 2012). In stromal cells lacking CAV1, upregulation of glycolytic enzymes such as aldolase A, PGK1, and PKM2, was shown, which indicated an inverse correlation between CAV1 and glycolysis (Pavlides et al., 2009). CAV1 deficiency can induce autophagy in HCC (Liu et al., 2016), while CAV1 was also reported to promote autophagy and inhibit apoptosis in breast cancer cells (Wang et al., 2014). Previously, the murine hepatocyte cell line AML12 was used to detect a metabolic switch from lipid to glucose metabolism (Fernandez-Rojo et al., 2012), but no gene expression analysis was conducted to provide a comprehensive view of CAV1 function in hepatocytes. In this work, delineating from pathway annotation analyses, only upregulated genes were correlating with different types of metabolic processes in primary hepatocytes lacking CAV1, and downregulated genes were related mainly to cancer-associated processes.

Transforming growth factor beta is one of the most studied cytokines in the liver. It is an important mediator of hepatic tissue repair, regeneration, and is associated with fibrogenesis (Dooley and Ten Dijke, 2012; Fernandez-Rojo et al., 2013; Meyer et al., 2013a; Fernandez-Rojo and Ramm, 2016; Li et al., 2017; Lu et al., 2018). Regarding metabolic events in liver pathophysiology, especially energy-generating processes such as glycolysis, TCA cycle, and oxidative phosphorylation, TGF-β functions are largely unexplored. Even though the role of TGF-β toward specific metabolic alterations has been investigated in some studies before, a comprehensive metabolic TGF-β gene signature in hepatocytes will now provide more in-depth insights, especially with focus on lipid metabolism (Yang et al., 2014), where now a detailed description of regulated targets is provided. Distinct metabolic signatures of TGF-β activation have been identified, which represent a critical step in unraveling the extent to which TGF-β may impact on biomolecule synthesis and catabolism, and also to determine pathophysiological outcome during disease progression. Indeed, although largely unappreciated in the context of TGF-β function, specific gene expression signatures of TGF-β were already shown in the study from Coulouarn et al. (2008). Accordingly, different time points were assessed to determine temporal TGF-β effects. At early time points, Smad co-repressors like *Skil*, *Tgif*, and the inhibitor *Smad7* were induced for providing negative feedback to TGF-β stimulation. Besides, some genes related to cell cycle arrest and apoptosis were also induced. At later time points, cell adhesion and matrix remodeling-related genes were altered, supporting the notion of a functional switch. Interestingly, metabolism-associated genes were also prominently suppressed, including lipid and redox homeostasis, cholesterol biosynthesis, and glutathione metabolism, indicating that metabolic genes were central targets of the TGF-β signaling network. These findings were consistent with our observation in primary hepatocytes (long-term *in vitro* treatment, 48 h). In conclusion, our results demonstrate the suppressive effect of TGF-β on metabolism in primary hepatocytes and also proved TGF-β as a crucial mediator of diverse pathways in cell pathology (Figure 2B). Importantly, with respect to responses to TGF-β stimulation, many metabolic genes outperformed commonly studied TGF-β target genes at the time point analyzed (48 h), such as *Smad6*, *Smad7*, and *Tgif*, likely because initial Smad signaling was reduced and secondary signaling events took over.

Due to the significance of the TGF-β pathway, TGF-β signaling is regulated at different levels. CAV1 particularly blocked TGF-β-induced cell death in hepatocytes and its expression level determined TGF-β functions in HCC cells (Meyer et al., 2013c; Moreno-Caceres et al., 2017). Intriguingly, CAV1 is overexpressed in various liver disease and HCC (but not in non-alcoholic fatty liver disease, own observations), suggesting that its expression may enable diseased hepatocytes to escape from detrimental TGF-β-induced responses (Nwosu et al., 2016; Moreno-Caceres et al., 2017). This hypothesis was supported by a previous study where the authors showed that CAV1 can inhibit TGF-β/Smad signaling to relieve liver fibrosis in mice (Lu et al., 2018). The entanglement between CAV1 and TGF-β was not only counteracting, because other data showed that both proteins work together to determine function. In an autophagy-impaired mouse model, Atg4b-deficient mice were treated with bleomycin to induce pulmonary fibrosis. Here, CAV1 expression was downregulated, while some TGF-β signaling-related genes were also downregulated, such as *Smad7* (Cabrera et al., 2015). Moreover, several genes were identified and their regulation by TGF-β seemed to be dependent on CAV1 level. Such genes are interesting research targets for upcoming studies with respect to TGF-β-CAV1 crosstalk in disease settings.

## Conclusion

In conclusion, we have provided gene expression signatures regulated by CAV1 and TGF-β in primary hepatocytes. Emphasis was put on the most prominently altered process, i.e., metabolism. The identification of metabolic gene signatures of TGF-β will facilitate the understanding of TGF-β control of liver metabolism, and could lead to better insights on how CAV1 and other proteins that block TGF-β may influence such control. Besides providing a new point of view for a deeper understanding on the entanglement from whole gene signatures in healthy hepatocytes, it will also be of relevance to investigate CAV1 and TGF-β correlation in disease settings, such as non-alcoholic steatohepatitis, cirrhosis, or HCC where CAV1 is often found to play a role. The presented microarray datasets will also be useful references for molecular profiling analyses, thus facilitating the search for potential novel targets in liver diseases.

## Data Availability Statement

Microarray data were deposited in GEO (https://www.ncbi.nlm.nih.gov/geo/) database (GSE137339).

## Ethics Statement

All animal procedures were performed according to ethical standards. Prior to experimentation, ethics approval was obtained from the local ethics committee of Baden-Württemberg.

## Author Contributions

MH and ZN performed the experiment work and wrote the manuscript. MH, ZN, and WP performed the microarray dataset analysis. ZN, SD, and ME proofread the manuscript. CM conceived the study, planned the experiments, and supervised the work. All authors read and approved the final version of the manuscript.

## Conflict of Interest

The authors declare that the research was conducted in the absence of any commercial or financial relationships that could be construed as a potential conflict of interest.

## Footnotes

1 https://www.ncbi.nlm.nih.gov/geo/

2 https://david.ncifcrf.gov/

3 http://bioinfogp.cnb.csic.es/tools/venny/

## Abbreviations

BMP: bone morphogenetic protein
CAV1: caveolin-1
FBS: fetal bovine serum
GEO: Gene Expression Omnibus
HCC: hepatocellular carcinoma
HFD: high-fat diet
JNK: c-JunN-terminal kinase
KEGG: Kyoto Encyclopedia of Genes and Genomes
KO: knockout
P/S: penicillin/streptomycin
PPIA: peptidylprolyl isomerase A
siRNA: small interfering RNA
TGF-β: transforming growth factor-beta
WT: wild type.
