# Supplementary material for: Caveolin-1 Impacts on TGF-β Regulation of Metabolic Gene Signatures in Hepatocytes
Source: Front Physiol. 2020 Jan 31;10:1606. doi: 10.3389/fphys.2019.01606 (PMC7005071; doi:10.3389/fphys.2019.01606)
Supplement: Supplementary file 2 [file Data_Sheet_1.pdf]

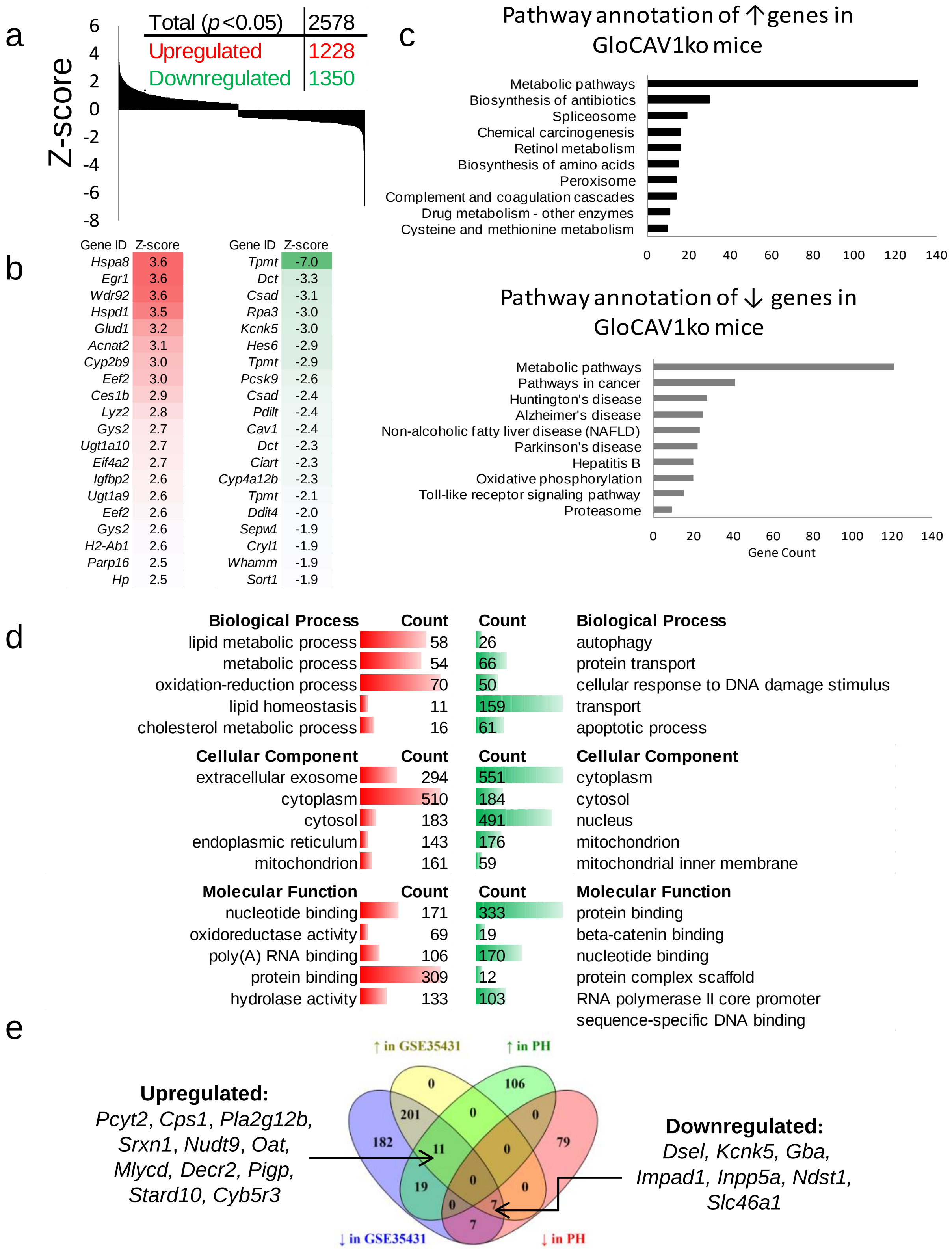
a
Z-score
6
4
2
0
-2
-4
-6
-8
Total (p<0.05) 2578
Upregulated 1228
Downregulated 1350
b
Gene ID Z-score
Hspa8 3.6
Egr1 3.6
Wdr92 3.6
Hspd1 3.5
Glud1 3.2
Acnat2 3.1
Cyp2b9 3.0
Eef2 3.0
Ces1b 2.9
Lyz2 2.8
Gys2 2.7
Ugt1a10 2.7
Eif4a2 2.7
Igfbp2 2.6
Ugt1a9 2.6
Eef2 2.6
Gys2 2.6
H2-Ab1 2.6
Parp16 2.5
Hp 2.5
Gene ID Z-score
Tpmt -7.0
Dct -3.3
Csad -3.1
Rpa3 -3.0
Kcnk5 -3.0
Hes6 -2.9
Tpmt -2.9
Pcsk9 -2.6
Csad -2.4
Pdilt -2.4
Cav1 -2.4
Dct -2.3
Ciart -2.3
Cyp4a12b -2.3
Tpmt -2.1
Ddit4 -2.0
Sepw1 -1.9
Cryl1 -1.9
Whamm -1.9
Sort1 -1.9
c
Pathway annotation of ↑genes in GloCAV1ko mice
Metabolic pathways
Biosynthesis of antibiotics
Spliceosome
Chemical carcinogenesis
Retinol metabolism
Biosynthesis of amino acids
Peroxisome
Complement and coagulation cascades
Drug metabolism - other enzymes
Cysteine and methionine metabolism
0 20 40 60 80 100 120 140
Pathway annotation of ↓ genes in GloCAV1ko mice
Metabolic pathways
Pathways in cancer
Huntington's disease
Alzheimer's disease
Non-alcoholic fatty liver disease (NAFLD)
Parkinson's disease
Hepatitis B
Oxidative phosphorylation
Toll-like receptor signaling pathway
Proteasome
0 20 40 60 80 100 120 140
Gene Count
d
Biological Process Count
lipid metabolic process 58
metabolic process 54
oxidation-reduction process 70
lipid homeostasis 11
cholesterol metabolic process 16
Count Biological Process
26 autophagy
66 protein transport
50 cellular response to DNA damage stimulus
159 transport
61 apoptotic process
Cellular Component Count
extracellular exosome 294
cytoplasm 510
cytosol 183
endoplasmic reticulum 143
mitochondrion 161
Count Cellular Component
551 cytoplasm
184 cytosol
491 nucleus
176 mitochondrion
59 mitochondrial inner membrane
Molecular Function Count
nucleotide binding 171
oxidoreductase activity 69
poly(A) RNA binding 106
protein binding 309
hydrolase activity 133
Count Molecular Function
333 protein binding
19 beta-catenin binding
170 nucleotide binding
12 protein complex scaffold
103 RNA polymerase II core promoter sequence-specific DNA binding
e
↑ in GSE35431
↑ in PH
↓ in GSE35431
↓ in PH
0
106
201
0
0
182
11
0
79
19
0
0
0
7
7
Upregulated:
Pcyt2, Cps1, Pla2g12b, Srxn1, Nudt9, Oat, Mlycd, Decr2, Pigp, Stard10, Cyb5r3
Downregulated:
Dsel, Kcnk5, Gba, Impad1, Inpp5a, Ndst1, Slc46a1

a

Gene	p-Value	Z-score
Cdkn2b	4.8E-07	4.2
Ltbp1	0.0004	2.3
Tgfbr1	1.30E-08	2.6
Smad7	1.5E-07	2.5
Acvr1	0.000026	2.0
Tgfb1	0.0004	1.6
Thbs1	0.0000044	1.0
Bmpr2	0.0003	1.1
Nbl1	0.0009	0.8
Tgif1	0.0001	0.6
Smad5	0.0085	0.4
Tgfbr2	0.0007	0.7
Bmpr1a	0.0001	0.6
Cul1	0.0016	0.4
Bambi	0.0366	0.2
Smad4	0.0014	0.3
Smad6	0.0007	0.3
Rhoa	0.0246	0.2

b

downregulated:
Idh1, Slc35c1, Grhpr, Hmbs, Slc25a13, Dhrs1, Sepx1, Naprt1, Pcyt2, Dgat2, Asns, Slc35d2, Kctd3, Lpin2, Slc26a1, Hmgcs1, Dhcr7, Entpd5, Abcc3, Dhcr24, Gss, Sc4mol, Sqle, Uap1l1, Chka, Scp2, Hpgd, Slc40a1, Gpd1, Psat1, Aldob, Gpt2, Aldh1a1

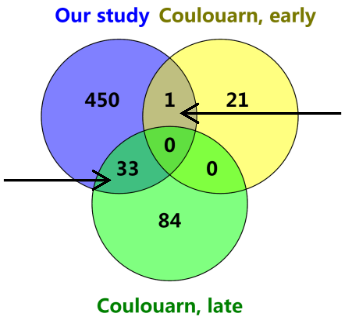

downregulated:
Aldh3a2

c

upregulated:
Rdh10, Gsto1, Slc6a8, Sult1e1, Lpgat1

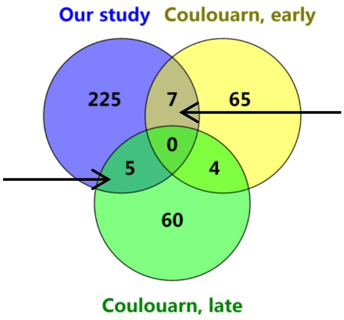

upregulated:
Slc25a37, Slc23a2, Clic4,Slc20a1,Qcnt2, Slc29a1, Coq10b
